# Factors shaping willingness to adopt generative artificial intelligence tools among university students: a TAM-based investigation

**DOI:** 10.3389/fpsyg.2026.1831505

**Published:** 2026-06-19

**Authors:** Wei Xuan Hu, Chu Yue Gao, Yu Xin Qin, Hai Yan Guo

**Affiliations:** School of Management, Tianjin University of Commerce, Tianjin, China

**Keywords:** behavioral intention, generative artificial intelligence platform, structural equation model, Technology Acceptance Model, university students

## Abstract

With the rapid advancement of generative artificial intelligence (GAI) technology, college students represent a crucial demographic in the adoption of emerging technologies. Their willingness to use such tools plays a significant role in shaping the integration and dissemination of GAI platforms. Extending the Technology Acceptance Model (TAM), this study proposes and tests a sequential mediation chain: perceived ease of use → perceived usefulness → satisfaction → user trust → behavioral intention. and develops a corresponding structural equation model (SEM) to systematically explore the factors influencing college students’ willingness to use GAI platforms. The study employed a questionnaire survey method and collected a total of 334 valid responses. Results confirm that perceived ease of use and perceived usefulness exert significant direct positive effects on satisfaction. Satisfaction, in turn, further enhances users’ trust in GAI platforms, and user trust is the key factor driving behavioral intention. This study enriches the theoretical application of the TAM in the context of GAI platforms, identifies key pathways influencing university students’ behavioral intentions, and has valuable practical implications for the design optimization, trust mechanism development, and formulation of promotion strategies for GAI platforms.

## 1 Introduction

The rapid advancement and widespread adoption of large language models (LLMs) and generative artificial intelligence (GAI) technologies have catalyzed the emergence of numerous GAI platforms—including ChatGPT, DeepSeek, Gemini, and Doubao. Recognized as a pivotal driver of new-quality productive forces, GAI is accelerating the convergence of digitalization, networking, and intelligence—fundamentally transforming how individuals produce, live, communicate, and learn. Especially, the explosive development of GAI technologies has become a particularly potent catalyst for educational change (Wen, 2026). Its rapid evolution is not merely augmenting existing practices but actively reshaping the structural foundations of the educational ecosystem. While it demonstrably enhances teaching efficiency and enables scalable, personalized learning, it also brings about multiple challenges such as ethical governance and educational equity (Ma and Peng, 2026).

Current research on the willingness to use GAI, both domestically and internationally, has primarily focused on technical aspects such as technical architecture, model optimization, multimodal integration, and human-computer interaction; at the user behavior level, scholars have largely relied on classical behavioral theories such as the Technology Acceptance Model (TAM) and the Theory of Planned Behavior (TPB) for explanatory analysis (Wu, 2007). However, empirical work situated specifically within educational contexts remains narrowly scoped, focusing chiefly on domain-specific instructional applications, prescriptive usage guidelines, and task-oriented learning support. Critically, there is a paucity of theory-driven, mechanism-oriented research that unpacks the psychological, contextual, and technological antecedents of college students’ GAI adoption decisions—and notably absent is a systematic comparative analysis of how students’ engagement with GAI differs from their use of conventional information retrieval tools. Therefore, this study aims to systematically investigate the mechanisms influencing college students’ willingness to use GAI in the educational sector, thereby promoting the deep empowerment and sustainable development of GAI in Chinese higher education.

This study aims to advance the deep integration and sustainable adoption of GAI platforms among China’s higher education population. In terms of theoretical significance, it extends the TAM by adapting it to the educational use of GAI—specifically through an empirical investigation of university students’ behavioral intentions. Furthermore, the theoretical uniqueness of GAI lies in its generative nature and output instability. Alongside its rapid development and widespread adoption, governance challenges related to data, information, and content urgently need to be addressed, necessitating a balance between technological advancement and safety (Ma and Wang, 2026). Consequently, trust in GAI systems is not only more consequential but also more susceptible to erosion than trust in traditional search engines. Therefore, compared with the traditional TAM, this study introduces “trust” as a key mediating construct and proposes a sequential mediation pathway: Perceived Ease of Use (PEU) → Perceived Usefulness (PU) → Satisfaction (ST) → User Trust (UT) → Behavioral Intention (BI). This full chain has remained empirically underexplored in prior research on GAI adoption in education, thus offering a novel theoretical contribution to understanding how college students form BI toward this emerging technology in academic contexts. It is important to clarify that issues specific to generative artificial intelligence (GAI)—such as AI hallucinations, algorithmic transparency, and ethical concerns—serve solely as the theoretical backdrop for this study. And the concept of trust has been incorporated into the empirical model. While these issues are explicitly discussed to underscore the centrality of user trust in GAI adoption, they have not been incorporated into the current empirical model. This is primarily because the absence of psychometrically validated measurement instruments, and this study focuses on the chained mediation mechanism of the core variables in the TAM. Consequently, these GAI-specific issues are reserved for the Discussion section, where they inform theoretical extensions and delineate concrete avenues for future empirical inquiry. In terms of practical significance, this study accurately matches users’ actual needs, effectively enhances users’ willingness to use GAI, and provides reference support for GAI developers and edtech enterprises in formulating optimization and improvement strategies. Within higher education, the study supports the cultivation of interdisciplinary GAI competencies and contributes to the systemic digital transformation and to the enhancement of teaching, learning, and research quality.

## 2 Literature review

### 2.1 TAM

The TAM is among the earliest theoretical frameworks employed to explain users’ willingness to adopt new technologies. It posits that technology acceptance is primarily influenced by two key factors: PU and PEU, which in turn shape users’ BI, often mediated by ST (Davis, 1989). In current research on the usage intention of GAI, scholars both at home and abroad have frequently drawn upon TAM and related theories, with a focus on user experience and ST. For instance, studies suggest that higher PU of GAI products is associated with stronger BI (Liu, 2024). The study primarily uses the TAM model to empirically examine how PU and PEU shape university students’ BI in adopting GAI in academic contexts. It further analyzes differences among college students in technology acceptance and usage intention, and explores the mechanisms by which GAI affects college students’ learning abilities. Previous scholars have used TAM as a theoretical framework to investigate differences among college students regarding perceived interactivity, PU, PEU, technology anxiety, and BI in relation to GAI, as well as the mechanisms underlying these differences (Wang and Yu, 2024; Chen and Zhang, 2026). Building upon TAM, an extended framework has been developed to show how GAI use specifically enhances business students’ capacity to formulate innovation- and entrepreneurship-oriented business plans (Hu et al., 2026). Empirical evidence confirms that GAI and LLMs are increasingly embedded in higher education practices, and their use in the educational field aligns with the TAM model’s ability to explain users’ subjective attitudes and behavioral tendencies toward new technologies.

### 2.2 User trust

In human-computer interaction, UT is a critical factor that determines the extent to which humans are willing to rely on and accept the recommendations and decisions of artificial intelligence systems (Yang, 2025). UT refers to users’ confidence in the reliability and safety of products and services (Wong et al., 2016). Trust is a core element in human-computer interaction, directly influencing the intensity and sustainability of such interactions (Xiang, 2024). Research has found that users’ level of trust in GAI significantly influences their willingness to use it, with functional trust (such as system reliability) having a greater impact on usage intent than human-like trust (such as anthropomorphism) (Choung et al., 2023). Scholars have pointed out that students need to be cautious about the risks posed by the generative nature and uncertainty of GAI, including algorithmic bias, technological dependence, information cocoons, and technology abuse (Zhou et al., 2026).

Currently, AI outperforms humans in areas such as pattern recognition and machine learning, but falls short of human intelligence in adaptability, ethical judgment, and explainability. It is a “tool” that can only “assist” rather than “replace” human intelligence. The phenomenon of AI hallucinations—factual errors or false citations caused by the output uncertainty and generative nature of generative artificial intelligence—has led to a crisis of trust among users regarding the generated content. Research suggests that user trust influences users’ usage attitudes and behavioral intentions, and points out that human-AI collaboration can be used to identify and correct errors, thereby reducing the probability of AI hallucinations and enhancing UT in GAI (Wang, 2026; Huynh and Aichner, 2025). In the GAI contexts, UT is inextricably contingent upon algorithmic transparency and the mitigation of hallucination risk. When outputs lack explanatory grounding and exhibit systematic inaccuracies, user trust will significantly decrease. Moreover, ethical concerns will weaken the effect of user trust on the willingness to use. This erosion stems fundamentally from the black-box nature of their algorithms: the internal mechanisms and decision pathways of large language models remain inherently uninterpretable to users, impeding human verification, undermining perceived credibility, and compromising functional reliability (Liu, 2025).

User perceptions of GAI constitute a primary antecedent of trust formation. Grounded in the TAM, integrating ‘UT’ as a core mediating construct—rather than a peripheral variable—enables more theoretically grounded and empirically precise analysis of the determinants shaping BI to adopt. Existing studies have highlighted distinct information-seeking strategies and exhibited differential cognitive processing patterns between users of GAI and traditional search engines. Research indicates that GAI demonstrates relative strength in the areas of emotionally resonant social interaction and career-oriented guidance, while traditional search engines retain stronger user-perceived credibility and depth in the academic domain (Wang et al., 2025; Niu et al., 2026).

Scholars both domestically and internationally have focused on three main areas regarding user trust: the connotations and dimensions of UT and their impact on BI; the trust crisis caused by the risks of AI hallucinations and ethical issues in GAI; and a comparative analysis of the differences in establishing UT between GAI and traditional search engines.

### 2.3 Literature review

Through a systematic review of domestic and international literature, the study points out the fundamental distinctions between GAI and traditional search engines—particularly in terms of output agency, contextual adaptability, and epistemic opacity. Moreover, the TAM model has been widely applied to discussions of user experience, ST, and related issues in GAI. However, emerging evidence indicates that UT functions not merely as an outcome but as a critical cognitive mediator in human-GAI interaction—especially when mitigating risks associated with GAI hallucinations, factual inconsistency, and ethical ambiguity. Yet existing TAM-based studies largely treat UT as an isolated construct or peripheral moderator, rather than integrating it structurally into the core acceptance pathway. Therefore, this study advances a theoretically grounded extension of the traditional TAM for educational GAI adoption and expands it to the context of educational adoption research. On this basis, “user trust” is introduced as an intermediary variable to expand the TAM model, and a chain of mediating paths of PEU → PU → ST → UT → BI is constructed to reveal the formation mechanism of usage intention of generative artificial intelligence in educational scenarios.

## 3 Research model and hypotheses

### 3.1 The influence of PEU on PU

Grounded in the Unified Theory of Acceptance and Use of Technology (UTAUT), PEU—defined as the degree to which using a technology is believed to be effortless—is theoretically established as a direct antecedent of PU (Venkatesh et al., 2003). This foundational relationship has been consistently validated in subsequent information systems research. Extending this premise to the context of GAI, empirical evidence confirms that when users perceive a GAI tool as easy to interact with, they are more likely to recognize its practical utility and value (Jo, 2022). Further supporting this causal chain, a recent study on technology adoption among students demonstrated that external facilitating conditions enhance PEU, which in turn exerts a direct and positive influence on PU (Xu et al., 2025). Thus, a coherent theoretical and empirical logic indicates that facilitating a user-friendly experience with GAI platforms strengthens perceptions of their usefulness. Based on this established causal pathway, the following hypothesis is proposed:

*H1:* PEU has a positive effect on PU.

### 3.2 The influence of PU and PEU on ST

PU is defined as the extent to which an individual believes that using a specific technology—in this context, GAI platforms for content creation—will enhance their performance or add value to their tasks. Grounded in established technology acceptance literature, a high level of PU, along with PEU, cultivates a more favorable evaluation of a technology, which is fundamentally captured by the construct of ST (Wang, 2025). This relationship is further theoretically anchored in post-adoption behavior research, which identifies ST as a direct cognitive outcome of PU, with a well-documented positive linkage between the two (Bhattacherjee, 2001). Extending this logic to integrated theoretical frameworks, studies combining user experience, TAM, and the expectation confirmation model (ECM) provide empirical evidence that PEU exerts a significant and positive influence on user ST (Guan, 2020). This pathway is corroborated within the specific domain of GAI; research validated through the Technology Acceptance Model 2 (TAM2) confirms that PEU, PU, and perceived enjoyment collectively and positively shape user ST (Song, 2025). Therefore, based on this cohesive theoretical and empirical foundation, the following hypotheses are advanced:

*H2:* PU positively influences user ST.

*H3:* PEU positively influences user ST.

### 3.3 The impact of ST on UT

The concept of trust has been extended and refined in information systems research, giving rise to a specialized dimension: “technological trust.” UT as a pivotal determinant of GAI adoption, has been quantitatively assessed in UT and ST with GAI, revealing a strong positive correlation between these variables (Li et al., 2025). The emotional responses of users often shape their level of trust in specific technologies (Lahno, 2001; Komiak and Benbasat, 2006). During AI interactions, positive emotions serve as salient heuristic cues along the heuristic processing path, enabling users to rapidly generate favorable responses, such as a heightened willingness to adopt the technology (Venkatesh, 2000; Tan et al., 2022). Building on the foregoing insights, perceived enjoyment in human-AI interactions serves to strengthen UT (Zhao et al., 2025). The following hypothesis is proposed:

*H4:* User ST has a positive influence on UT.

### 3.4 The influence of PEU and PU on UT

In the AI context, trust is defined as the belief that the services and outputs generated by AI agents are reliable and trustworthy (Shin, 2021). People believe that PEU will increase UT in information systems (Sarkar et al., 2020). Previous studies have shown that PEU positively influences trust (Liu and Tao, 2022; Li et al., 2023). Relevant empirical evidence further demonstrated that, for AI chatbots, factors such as PEU and anthropomorphism positively affect UT (Mogaji et al., 2021). A meta-analysis of 65 published empirical studies confirmed that PU, PEU, and product performance are crucial antecedents of UT (Zhang et al., 2025). Based on this body of evidence, the following hypotheses are posited:

*H5:* PEU has a positive influence on UT.

*H6:* PU has a positive influence on UT.

### 3.5 The impact of UT on BI

BI denotes the individual’s conscious decision to adopt or continue using a specific technology, system, or software, shaped by PU and PEU—two core antecedents in the TAM, and serves as a proximal predictor of actual usage behavior. When using artificial intelligence, users perceive that problem-solving becomes easier or more efficient, which enhances their trust in AI and subsequently increases their BI (Lee et al., 2023). Research has demonstrated a strong correlation between UT and BI (Fu et al., 2023). The model was empirically validated using survey data collected from 632 participants. The results revealed a mediating effect of trust whereby UT can transform static basic characteristics into positive BI toward AI adoption and use (Zhao et al., 2025). Accordingly, the following hypothesis is assumed:

*H7:* UT has positive influence on BI.

**Figure 1:**
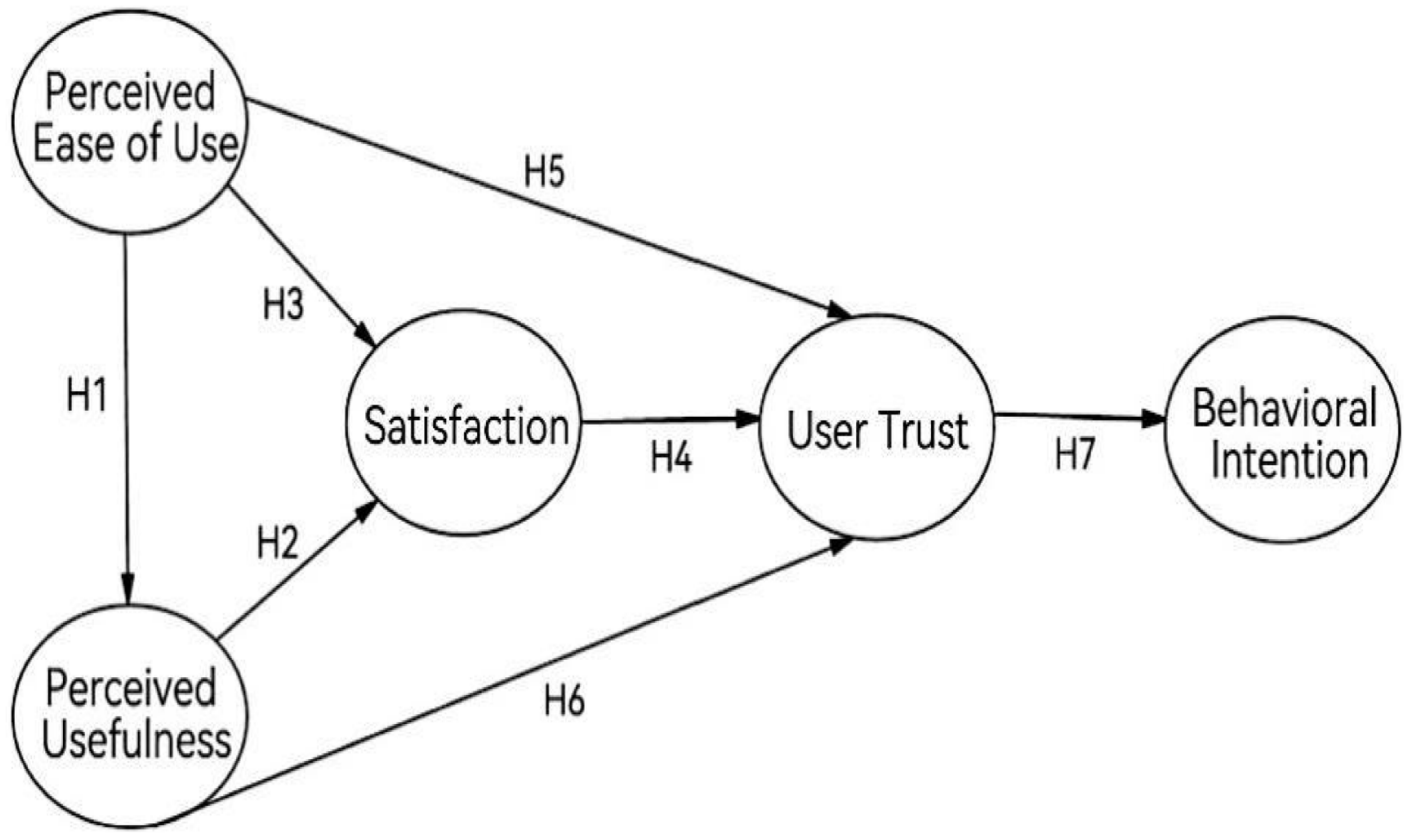
Research model.

Based on the above analysis, this study adopts the TAM as its theoretical foundation, incorporating PEU and PU as antecedent variables, ST and UT as mediating variables, and BI as the outcome variable. It systematically investigates the influencing factors and their pathways in shaping college students’ willingness to use GAI platforms. The preliminary research framework is illustrated in Figure 1.

## 4 Methods

### 4.1 Questionnaire design

To examine the five constructs of the TAM model used in this study, we drew upon the scale items from previous research. We systematically adapted validated scale items from established empirical and theoretical sources. For PEU, we used the scale adapted by Lakhal et al. (2013) based on Venkatesh et al. (2003), example item: “Learning to operate Elluminate will be easy for me.” Additional items were drawn from Venkatesh and Davis (2000) theoretical refinement of TAM, including “I find it easy to get the system to do what I want it to do.” PU dimension was measured using parallel items from the same source (Venkatesh and Davis, 2000). For instance, “Using the system improves my performance in my job.” For the ST dimension, this study adopted the overall ST scale adapted by Bhattacherjee (2001) from Spreng et al. (1996), representative item: “I’m very satisfied with my overall experience of OBD.” Hussain and Khan (2025) adapted the items based on the scale proposed by Yoon (2016), such as “I’m satisfied that AI in my library meets my needs.” For UT, this study drew upon the scale developed by Chen et al. (2024) based on prior literature, such as “The information provided by ChatGPT is comprehensive.” It also references the scale modified by Hyun Baek and Kim (2023) based on Kim and Baek (2022), such as “ChatGPT is trustworthy.” Finally, BI was operationalized using items from Venkatesh and Davis (1996) seminal TAM validation study (e.g., “Assuming I had access to WordPerfect, I intend use it”) and further supplemented with an item adapted by Hariri and Roberts (2015) from Venkatesh et al. (2003) reflecting BI toward emerging learning technologies: “I intend to use a learning innovation in the near future.”

To align the measurement framework with the generative AI learning context of this study—and to reflect its defining technical attributes, including intelligent content generation, interactive human-AI collaboration, and cognitive augmentation—we reframed the theoretical grounding beyond traditional information-based educational software and conventional office productivity systems. Consequently, scale items inconsistent with the core research context—namely, college students’ authentic use of GAI to support learning activities, enhance learning efficiency, and improve academic performance—were systematically excluded. The target construct in all retained items is GAI (e.g., “Elluminate,” “system,” or “OBD”), not generic software or abstract systems. Based on this, this study has designed a scale with 5 variables, as shown in Table 1.

**Table 1:** Scale items and sources of indicators.

Constructs	Symbol description	Indicators	Sources
Perceived ease of use (PEU)	PEU1	I think it’s easy to get in touch with and use generative artificial intelligence.	Lakhal et al. (2013) and Venkatesh and Davis (2000)
	PEU2	I have a good command of generative artificial intelligence and can interact with it clearly and understandably.	
	PEU3	I found it quite easy to make the generative artificial intelligence operate according to my wishes.	
Perceived usefulness (PU)	PU1	I believe that using artificial intelligence would improve my job performance.	Venkatesh and Davis (2000)
	PU2	I believe that using generative artificial intelligence can enhance my efficiency in work, study, and daily life.	
	PU3	I think the content generated by artificial intelligence is valuable.	
Satisfaction (ST)	ST1	I find using generative artificial intelligence very pleased.	Bhattacherjee (2001) and Hussain and Khan (2025)
	ST2	I’m completely satisfied with the performance of generative artificial intelligence.	
	ST3	I believe that generative artificial intelligence can fully meet my needs.	
	ST4	I believe that generative artificial intelligence is extremely useful for both students and teachers in schools.	
User trust (UT)	UT1	Generative artificial intelligence is comprehensive.	Venkatesh and Davis (1996) and Hariri and Roberts (2015)
	UT2	Generative artificial intelligence is credible.	
	UT3	Generative artificial intelligence is trustworthy.	
Behavioral intention (BI)	BI1	Assuming I have access to generative artificial intelligence, I intend to use it.	Chen et al. (2024) and Hyun Baek and Kim (2023)
	BI2	I predict I would use generative artificial intelligence in the near future.	
	BI3	I plan to continue using generative artificial intelligence in the near future.	

### 4.2 Sample and data collection

The research sample consisted of university students from diverse regions across China. Meanwhile, we obtained oral informed consent from all subjects to participate in the research. To minimize misinterpretation during data collection, we supplemented the data collection with explanatory annotations and auxiliary instructions and assured respondents of privacy protection, thereby alleviating concerns and enhancing data reliability. Through sample attributes such as gender, grade, and major, the target population was accurately represented. The survey was administered in December 2025 and remained open for 2 months, concluding in January 2026. A total of 352 samples were obtained after the questionnaires were closed for retrieval. Invalid data were excluded based on criteria such as incomplete responses or noncompliant formats, including questionnaire completion times under 60 s, consistent lack of variation in scale responses, or persistent extreme values. In the end, 334 valid questionnaires were obtained with an overall sample validity rate of approximately 94.9%. In the sample, males accounted for 51.2%, and females accounted for 48.8%. The grade level was mainly concentrated in the second year of university. In terms of academic majors, science-related majors accounted for a relatively large proportion (35.33%). Regarding usage frequency, the largest number of participants (117) reported using it 61–100 times per week. Table 2 shows the demographic details of the 334 participants.

**Table 2:** Demographic profile of respondents.

Statistical variables	Category	Frequency	Percentage (%)
Gender	Male	171	51.20
	Female	163	48.80
Grade	Freshman	43	12.87
	Sophomore	89	26.77
	Junior	58	17.26
	Senior	67	20.15
	Postgraduate	77	21.95
Major	Humanities	89	26.65
	Sciences	118	35.33
	Arts	68	20.36
	Economics and management	47	14.07
	Others	12	3.60
Frequency of use (per week)	0 ~ 5 times	13	3.90
	6 ~ 20 times	36	10.78
	21 ~ 60 times	75	22.46
	61 ~ 100 times	117	35.03
	more than 100 times	93	27.84

### 4.3 Analysis method

This study used SPSS 27.0 for descriptive statistics, reliability analysis, Harman’s single-factor test, correlation analysis and hierarchical regression. AMOS 29.0 was used for confirmatory factor analysis (CFA) to evaluate convergent validity, discriminant validity and model fit indices. SmartPLS 4 was applied for structural equation modeling (SEM), path analysis, mediation effect testing, predictive relevance (Q^2^) and multi-group robustness analysis.

SEM is a multivariate statistical method that analyzes relationships based on their covariance matrix. It is often used for confirmatory factor analysis, higher-order factor analysis, path analysis, and causal analysis, among other applications. This study establishes a TAM-SEM framework to investigate the factors influencing college students’ BI toward generative AI platforms. To strictly assess the model’s goodness-of-fit and applicability, the model is subsequently tested using mediation tests, hierarchical regression, predictive relevance, and robustness check.

## 5 Empirical analysis

### 5.1 Survey data processing

#### 5.1.1 Harman’s single-factor test

To address potential common-method bias, we conducted Harman’s single-factor test by performing an exploratory factor analysis (EFA) of all scale items without rotation and constraining the solution to a single factor. The results in Table 3 show that the first common factor explained 32.583% of the variance, which is below the critical threshold of 50% (Podsakoff and Organ, 1986). This indicates that there is no serious common-method bias in the data of this study.

**Table 3:** Harman’s single factor.

Component	Initial eigenvalues			Extract the sum of squares and load			Rotate and load the square sum		
	Total	% of variance	Cumulative %	Total	% of variance	Cumulative %	Total	% of variance	Cumulative %
1	5.256	32.853	32.853	5.256	32.853	32.853	2.697	16.854	16.854
2	1.812	11.327	44.18	1.812	11.327	44.18	2.193	13.703	30.558
3	1.519	9.494	53.674	1.519	9.494	53.674	2.181	13.631	44.189
4	1.409	8.805	62.479	1.409	8.805	62.479	2.126	13.285	57.474
5	1.297	8.104	70.583	1.297	8.104	70.583	2.098	13.11	70.583
6	0.553	3.455	74.038	–	–	–	–	–	–
7	0.521	3.257	77.294	–	–	–	–	–	–
8	0.497	3.103	80.398	–	–	–	–	–	–
9	0.463	2.893	83.29	–	–	–	–	–	–
10	0.442	2.765	86.056	–	–	–	–	–	–
11	0.431	2.691	88.747	–	–	–	–	–	–
12	0.409	2.556	91.303	–	–	–	–	–	–
13	0.384	2.402	93.704	–	–	–	–	–	–
14	0.376	2.35	96.054	–	–	–	–	–	–
15	0.34	2.122	98.176	–	–	–	–	–	–
16	0.292	1.824	100	–	–	–	–	–	–

Variance explained is a metric that measures a model’s ability to account for variation in the data and is commonly used in statistical analyses such as regression and factor analysis. It reflects the model’s explanatory power with respect to data variability, with higher values indicating greater explanatory capability. This study extracted five common factors with eigenvalues exceeding 1. As shown in Table 3, both the cumulative variance explained before rotation and after varimax orthogonal rotation were 70.583%, indicating that the model adequately describes the information in the original variables. Furthermore, the rotated factor solution underlying the structural relationships among variables is relatively balanced.

#### 5.1.2 Reliability analysis

In evaluating the measurement model, it is necessary to ensure its reliability and validity. First, the test results for Cronbach’s alpha are shown in Table 4. Cronbach’s *α* is one of the widely used indicators of internal consistency in scales and questionnaires, with values ranging from 0 to 1. Higher values indicate greater internal consistency (Spiliotopoulou, 2009). The results show that all scales achieved Cronbach’s α coefficients exceeding 0.77, demonstrating overall satisfactory reliability. This supports the reliability of the measurement model and ensures stable and consistent data quality. The composite Cronbach’s α for the entire questionnaire reached 0.863, further confirming its high reliability.

**Table 4:** Reliability measures.

Constructs	Number of terms	Cronbach’s alpha
PEU	3	0.777
PU	3	0.807
ST	4	0.833
UT	3	0.812
BI	3	0.788
Total	16	0.863

#### 5.1.3 Validity analysis

The Kaiser–Meyer–Olkin (KMO) test evaluates the relative magnitude of the original variables compared to simple and partial correlation coefficients. A value closer to 1 indicates stronger inter-variable correlations, suggesting greater suitability of the variables. As shown in Table 5, the KMO measure of sampling adequacy is 0.851, and Bartlett’s test of sphericity yielded a *p*-value <0.001, collectively supporting the presence of adequate intercorrelation and shared variance among the variables, thereby justifying the use of exploratory factor analysis.

**Table 5:** KMO and Bartlett tests.

Test category	Test statistics	Value
Kaiser-Meyer-Olkin measure of sampling		0.851
Bartlett’s sphericity test	Approximate Chi-square	2039.217
	df	120
	*P* value	0.000

The factor loading coefficient represents the weight of each original variable in the factor expression, indicating the degree to which the extracted common factor influences the original variable. As shown in Table 6, the rotated component matrix indicates that all factor loadings for each test-type factor exceed the 0.5 threshold, demonstrating a strong association between the variables and the factors. Overall, the factor structure is clear, suggesting that the questionnaire scale exhibits good structural validity in terms of dimension division.

**Table 6:** Rotated constituent matrix.

Variables	Item	Factor loading coefficient				
		Component 1	Component 2	Component 3	Component 4	Component 5
PEU	PEU1					0.831
	PEU2					0.779
	PEU3					0.776
PU	PU1			0.786		
	PU2			0.825		
	PU3			0.807		
ST	ST1	0.806				
	ST2	0.760				
	ST3	0.816				
	ST4	0.772				
UT	UT1		0.766			
	UT2		0.825			
	UT3		0.844			
BI	BI1				0.821	
	BI2				0.793	
	BI3				0.778	

To ensure the model’s reliability and validity, this study also conducted assessments of convergent validity and composite reliability. Convergent validity is supported when the average variance extracted (AVE) exceeds 0.5, and composite reliability (CR) is a widely adopted criterion in structural equation modeling and scale validation studies (Hew et al., 2017; Flavián et al., 2022). The results presented in Table 7 show that all latent variables have AVE values greater than 0.5 and CR values exceeding 0.6. The numerical results confirm that the model is well-validated.

**Table 7:** Convergent validity.

Variable	Measure item	Standard load coefficient	AVE	CR
PEU	PEU1	0.754	0.539	0.778
	PEU2	0.771		
	PEU3	0.675		
PU	PU1	0.777	0.583	0.807
	PU2	0.748		
	PU3	0.764		
ST	ST1	0.772	0.557	0.834
	ST2	0.721		
	ST3	0.784		
	ST4	0.704		
UT	UT1	0.756	0.554	0.788
	UT2	0.740		
	UT3	0.735		
BI	BI1	0.734	0.594	0.814
	BI2	0.766		
	BI3	0.811		

Discriminant validity refers to the ability to distinguish between observed values when different methods are used to measure different constructs. Discriminant validity is established when the square root of the AVE exceeds the correlations between variables, and when each indicator has a high loading on its respective variable—specifically, coefficients greater than 0.7 (Wong et al., 2016). As shown in Table 8, the square roots of the AVE for all five variables in the model exceed 0.7, providing strong evidence of discriminant validity.

**Table 8:** The discriminant validity.

Construct	PEU	PU	ST	UT	BI
PEU	0.734				
PU	0.320	0.763			
ST	0.283	0.330	0.746		
UT	0.347	0.391	0.317	0.771	
BI	0.356	0.349	0.341	0.326	0.744

Bold values indicate the square root of AVE.

#### 5.1.4 Correlation analysis

The coefficient of determination (*R*^2^) is employed to assess the accuracy of model predictions. R2 ranges from 0 to 1, with higher values indicating greater explanatory power. Specifically, *R*^2^ values of 0.75, 0.50, and 0.25 are generally interpreted as representing substantial, moderate, and weak levels of predictive accuracy, respectively (Hair et al., 2017). As shown in Table 9, all latent variables demonstrate significant positive correlations, reflecting their strong theoretical linkages.

**Table 9:** Correlation analysis.

Construct	PEU	PU	ST	UT	BI
PEU	1				
PU	0.320**	1			
ST	0.283**	0.330**	1		
UT	0.347**	0.391**	0.317**	1	
BI	0.356**	0.349**	0.341**	0.326**	1

* *p* < 0.1; ** *p* < 0.05; *** *p* < 0.01.

### 5.2 Model verification and fit accuracy

#### 5.2.1 Model fit degree

The root mean square error of approximation (RMSEA) assesses the discrepancy between the hypothesized model and the population covariance matrix, reflecting the degree of model misfit per degree of freedom. RMSEA value less than 0.05 indicates an excellent model fit; values between 0.05 and 0.08 suggest an acceptable fit; and values between 0.08 and 0.10 indicate a moderate fit (MacCallum et al., 1996).

The comparative fit index (CFI) and the Tucker-Lewis index (TLI) range from 0 to 1, with values closer to 1 indicating better fit. Generally, a CFI value above 0.9 is considered acceptable for model fit; values above 0.95 indicate good model fit (Hu and Bentler, 1999). In this study, all fit indices meet or exceed the recommended thresholds, as shown in Table 10. This indicates that the proposed structural equation model has a good overall fit and explanatory power.

**Table 10:** Model fit degree.

Indicators	CMIN/DF	RMSEA	IFI	TLI	CFI	AGFI	GFI
Actual measurement results	1.449	0.037	0.978	0.972	0.978	0.931	0.951

#### 5.2.2 Mediation effect test

As illustrated in Table 11, the serial mediating path from PEU via PU, ST, and UT to BI demonstrates a significant positive effect. The indirect impact is 0.013, with a 95% confidence interval (CI) of [0.004, 0.027] that does not include 0, and the corresponding *p* value is 0.001 (*p* < 0.01), confirming the significance of this chain mediation. This indicates that PEU can enhance UT by improving PU, thereby strengthening UT and ultimately increasing BI.

**Table 11:** Mediation test.

Paths	Estimate	Lower	Upper	*P*
PEU- > PU- > ST- > UT- > BI	0.013	0.004	0.027	0.001
PEU- > UT- > BI	0.140	0.067	0.220	0.002
PU- > UT- > BI	0.123	0.048	0.208	0.001

Additionally, the individual mediating path of PEU and PU acting on the BI through UT also shows a positive effect. The mediating effects are all significant (*p* < 0.01), indicating that UT partially mediates the impact of PEU and PU on BI.

#### 5.2.3 Path verification

As shown in Table 12, PEU (*β* = 0.399, *p* < 0.001) has a considerable and favorable effect on PU. PEU (*β* = 0.232, *p* < 0.01) and PU (*β* = 0.312, *p* < 0.001) also had a significant and substantial impact on ST. Moreover, PEU (*β* = 0.302, *p* < 0.001), PU (*β* = 0.265, *p* < 0.001), and ST (*β* = 0.223, *p* < 0.01) exerted a significant positive effect on UT. In turn, UT (*β* = 0.465, *p* < 0.001) exerts a significant positive influence on BI. All paths reached statistical significance, indicating that the proposed relationships among the variables are robust and clearly directional. Furthermore, according to conventional benchmarks for interpreting effect sizes in structural equation modeling, an f2 value of ≥ 0.02 indicates a small effect, ≥ 0.15 a medium effect, and ≥ 0.35 a large effect. All the path effect sizes are greater than the standard of a small effect (≥ 0.02), indicating that each path variable has a substantive co-occurrence with the explained variable, and there are no meaningless path relationships. Specifically, PEU exerts a strong explanatory effect on PU (f^2^ = 0.117), and the effect size of UT on BI is 0.124, both of which contribute meaningfully to the model’s explanatory power.

**Table 12:** Result of structural model examination.

Hypotheses	Paths			Path coefficients	SE	CR	f^2^	*P*
H1	PU	<---	PEU	0.399	0.092	5.452	0.117	***
H2	ST	<---	PU	0.232	0.087	3.167	0.040	0.002
H3	ST	<---	PEU	0.312	0.069	4.271	0.076	***
H4	UT	<---	ST	0.302	0.085	4.029	0.060	***
H5	UT	<---	PEU	0.265	0.067	3.580	0.045	***
H6	UT	<---	PU	0.223	0.067	3.191	0.049	0.001
H7	BI	<---	UT	0.465	0.072	6.390	0.124	***

* *p* < 0.1; ** *p* < 0.05; *** *p* < 0.01.

#### 5.2.4 SEM result

**Figure 2:**
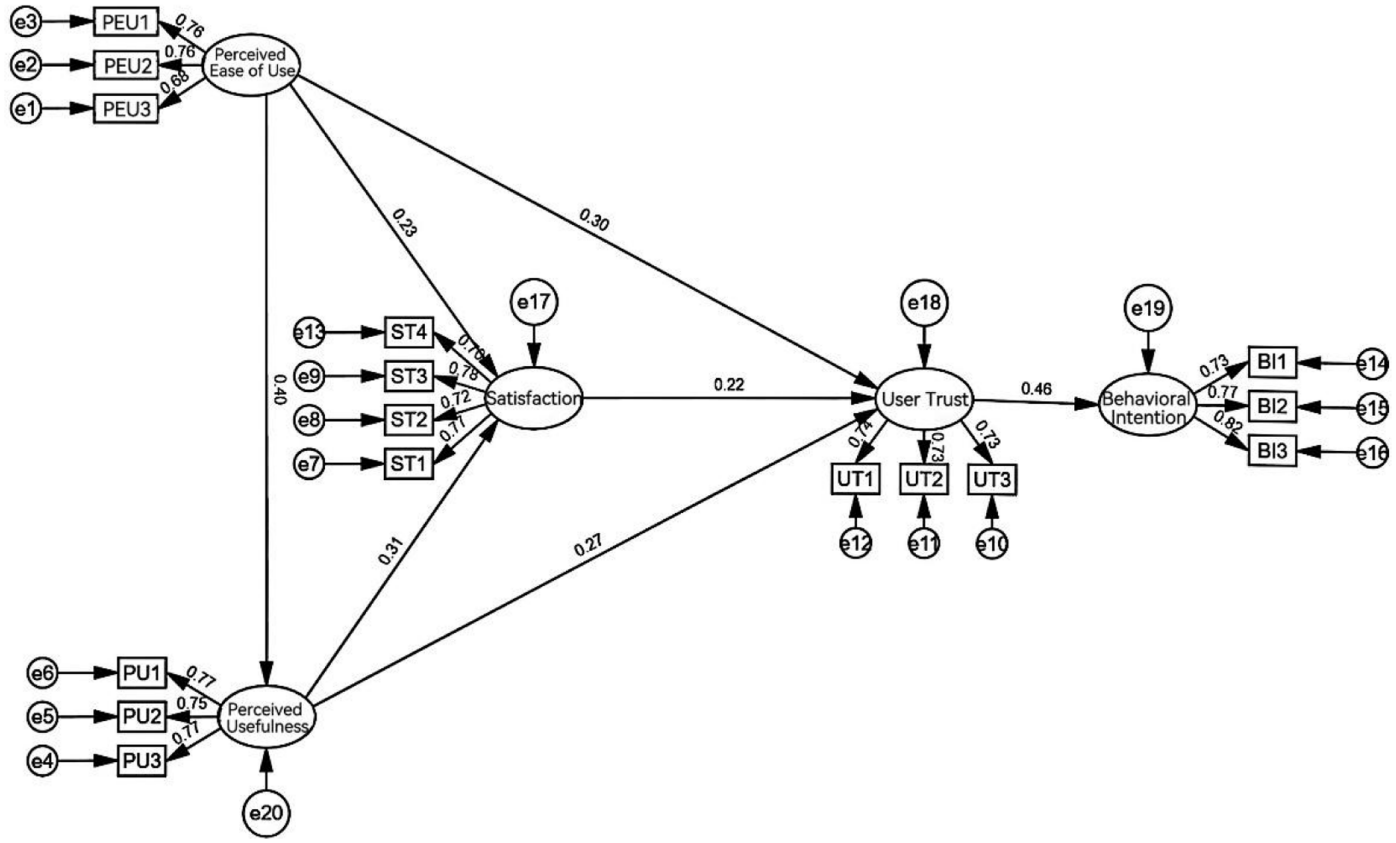
Model path coefficient diagram.

The SEM results indicate that all proposed paths were statistically significant at the *p* < 0.05 level. Notably, PEU exerted a robust positive effect on PU (*β* = 0.40, *p* < 0.001), and UT demonstrated a similarly strong positive influence on BI (*β* = 0.46, *p* < 0.001). All seven primary hypotheses H1, H2, H3, H4, H5, H6, and H7 were all supported. Furthermore, three distinct mediation pathways achieved full statistical support: PEU → PU → ST → UT → BI; PEU → UT → BI; and PU → UT → BI. The final SEM of this study is shown in Figure 2.

PEU and PU exert complementary and statistically significant effects on ST among Chinese college students using the GAI. SEM reveals that the standardized path coefficient of PEU on ST is 0.23 (*p* < 0.01), while that of PU is 0.31 (*p* < 0.001). Within PEU, the ease of initial contact, the simplicity of operation, and the clarity of output content collectively lower cognitive and behavioral barriers to GAI adoption. Meanwhile, PU by accurately meeting user needs further contributes to ST enhancement. Therefore, GAI platforms should prioritize improving output readability and practicality, expanding accessibility, providing tailored applications for university contexts and offering personalization, thereby enhancing overall user ST.

The path coefficient from ST to UT is 0.22 (*p* < 0.01), and that from UT to BI is 0.46 (*p* < 0.001), confirming a significant serial mediation pathway. This result indicates that user ST not only reinforces users’ cognitive appraisal of the GAI platform’s credibility and dependability but also increases usage frequency. This means that when the platform delivers timely, task-appropriate outputs with demonstrable reliability across diverse learning contexts, it is part of the learning process. Consequently, GAI platform developers should prioritize pedagogically validated core usage scenarios (e.g., academic writing support, conceptual explanation, code generation for STEM courses), standardize output formats and depth according to disciplinary conventions and educational levels (e.g., undergraduate vs. graduate), and enhance the role of GAI as an auxiliary tool in the education field.

#### 5.2.5 Hierarchical regression analysis

To systematically distinguish the effects of basic control variables from those of core research variables, and to accurately examine the causal pathways and incremental contributions among variables, this study also employed hierarchical regression analysis. By entering different sets of variables in a stepwise manner, the model fit and coefficient significance of the regression models were progressively compared. After controlling for other variables, the change in model performance attributable to the independent variable on the dependent variable was assessed.

First, we assess whether the selected variables exhibit severe multicollinearity, as such collinearity could bias the regression estimates and undermine the validity of our hypothesis tests. This study computes the Variance Inflation Factor (VIF) for each explanatory variable; a VIF value below 10 is conventionally regarded as indicating the absence of problematic multicollinearity. As shown in Table 13, the VIF values for all variables are well below 2—substantially lower than the commonly accepted threshold. Having passed the multicollinearity test, this indicates that none of the variables in the model used for hypothesis testing in this study exhibit severe multicollinearity.

**Table 13:** Hierarchical regression results.

Variable	Model 1			Model 2		
	B	*t*	VIF	B	*t*	VIF
Constant	3.225**	13.594***	–	1.242**	2.349*	–
Gender	−0.026	−0.285	1.010	−0.018	−0.297	1.014
Grade	0.006	0.134	1.003	0.033	0.685	1.017
Major	0.042	0.965	1.009	0.055	1.339	1.012
Frequency of use (per week)	0.111	1.930	1.004	0.092	1.652	1.015
PEOU	–			0.190***	3.372***	1.257
PU	–			0.239***	4.629***	1.259
ST	–			0.152**	2.604**	1.227
UT	–			0.129*	2.258*	1.298
R2	0.015			0.257		
Adjust R2	0.003			0.238		
△R2	0.015			0.208		
*F* value	1.224			15.527***		

Dependent variable = BI; * *p* < 0.05; ** *p* < 0.01; *** *p* < 0.001.

This hierarchical regression analysis involved two models. Model 1 included gender, grade level, major, and frequency of use as control variables. In contrast, Model 2 added PEU, PU, ST, and UT as core independent variables to Model 1. The dependent variable in both models was “willingness to use,” and the analysis utilized data from the questionnaire item “University students’ willingness to use generative artificial intelligence.”

As shown in Table 13, the *R*^2^ value of Model 1 is 0.015, indicating that basic demographic attributes account for only 1.5% of the variance influencing university students’ use of GAI. The F-test for Model 1 was non-significant (*F* = 1.224, *p* > 0.05), suggesting that the demographic characteristics included in the model do not collectively exert a statistically significant influence on students’ willingness to adopt GAI. For Model 2, after adding the factors influencing university students’ willingness to use GAI, as in Model 1, the *R*^2^ increased from 0.015 to 0.257, and the overall model was significant (*F* = 15.527, *p* < 0.001). This indicates that the four factors are PEU, PU, ST, and UT. Collectively, these four factors explain 25.7% of the variance in university students’ willingness to use GAI. The incremental *R*^2^ (Δ*R*^2^ = 0.208) attributable to the addition of these core independent variables is statistically significant, accounting for an additional 20.8% of the explained variance and underscoring their substantial incremental contribution beyond demographics. Specifically, PEU (*β* = 0.190, *t* = 3.372, *p* < 0.001), PU (*β* = 0.239, *t* = 4.629, *p* < 0.001), and ST (*β* = 0.152, *t* = 2.604, *p* < 0.01) all showed significant positive effects on university students’ willingness to use GAI.

#### 5.2.6 Predictive relevance test

Predictive relevance, as quantified by the Q^2^ statistic, serves as a key indicator of a SEM’s out-of-sample predictive power. If the Q^2^ value of the latent variable is greater than 0, it indicates that the model has a meaningful predictive relevance for that variable. As presented in Table 14, the Q^2^ values of all variables are significantly greater than 0, confirming that the path model not only fits the observed data well but also demonstrates acceptable predictive relevance.

**Table 14:** Predictive relevance test.

Variable	Q^2^
PEU	0.375
PU	0.428
ST	0.437
UT	0.390
BI	0.431

#### 5.2.7 Robustness check

To assess the invariance of path coefficients across demographic subgroups and mitigate potential bias arising from sample heterogeneity, this study conducted a multi-group analysis (MGA) stratified by gender. The results are presented in Table 15. Across the two groups (male and female), all core paths exhibited consistent directional effects and statistical significance. The *p*-values for the inter-group difference tests for all paths revealed greater than 0.05, indicating no significant differences between groups. These findings demonstrate that the proposed model shows acceptable stability across gender groups. The results should be generalized with caution, as robustness checks are limited to gender subgroup analysis in this study.

**Table 15:** Multiple group analysis.

Paths			Male (*n* = 171)		Female (*n* = 163)		Inter-group difference test
			*β*	*p*	*β*	*p*	*p*
PU	<---	PEU	0.271	0.000***	0.383	0.000***	0.262
ST	<---	PU	0.217	0.004**	0.325	0.000***	0.298
ST	<---	PEU	0.228	0.005**	0.160	0.035*	0.538
UT	<---	ST	0.170	0.019*	0.253	0.000***	0.396
UT	<---	PEU	0.322	0.000***	0.160	0.024*	0.105
UT	<---	PU	0.237	0.001**	0.174	0.028*	0.559
BI	<---	UT	0.400	0.000***	0.286	0.000***	0.201

* *p* < 0.05; ** *p* < 0.01; *** *p* < 0.001.

## 6 Discussion

This study develops a SEM based on the extended TAM to elucidate the formation mechanism of college students’ willingness to use GAI platforms. It successfully analyzes and verifies two consecutive influence paths: “PEU → ST → UT → BI” and “PU → ST → UT → BI,” thereby systematically clarifying how cognitive and social factors jointly shape BI.

Empirical results confirm that PEU has a significant positive impact on PU, ST, and UT, findings fully aligned with the hypothesized relationships. Similarly, PU significantly enhances both ST and UT. Crucially, ST serves as a key mediator in the pathways linking PEU and PU to UT: it not only transmits the influence of these antecedents but also significantly predicts UT itself. Moreover, a positive user experience mitigates trust concerns stemming from AI hallucinations, inaccuracies in generated content, and algorithmic opacity—thereby reinforcing trust formation. UT emerges as the strongest predictor of BI to use GAI platforms; in educational scenarios where generated content involves uncertainty, UT becomes the pivotal factor determining continued use. The chain-mediation pathway validated in this study—“PEU—PU—ST—UT—BI”—elucidates the stepwise progression from initial technology perception to concrete usage commitment, encompassing cognitive and affective processes. It addresses the shortcomings of the traditional TAM model, which largely overlooks trust dynamics in learning-oriented GAI applications, thereby uncovering the nuanced psychological mechanisms underlying GAI adoption among college students.

Currently, large-scale GAI models serve as key enablers across a wide range of human activities, including natural language processing and multimodal interaction. To maximize their educational value, GAI platforms should simplify interactions and lower the learning threshold, strengthen the functional practicality aligned with academic needs, and improve trust mechanisms through stable outputs, transparent algorithms, and content standardization. Universities, in turn, should strategically integrate GAI into digital pedagogy—not merely as a supplementary tool, but as an embedded component of curriculum design, faculty development, and student assessment—while establishing institution-wide guidelines for responsible, pedagogically grounded usage to advance the authentic integration of GAI and higher education.

This study foregrounds the theoretical significance of GAI-specific constructs—including AI hallucinations, algorithmic transparency, and ethical constraints—while explicitly positioning them as conceptual boundary conditions rather than empirically testable variables. In contrast, UT dimensions were formally incorporated into the research model and subjected to empirical testing. By identifying and empirically substantiating key distinctions in trust formation between generative AI systems and conventional information systems, this study establishes a foundational theoretical framework for future research on human–GAI interaction. To advance this line of inquiry, subsequent studies should develop psychometrically sound measurement instruments for constructs such as algorithmic transparency, hallucination susceptibility, and ethical alignment; validate their dimensional structure and cross-context reliability; and integrate them—as antecedents, mediators, or moderators—into extended empirical models. Such efforts will not only deepen theoretical understanding but also enable more nuanced, context-sensitive modeling of user cognition and behavior in GAI environments.

The application scope of future GAI platforms is projected to broaden continuously, while their user base grows increasingly heterogeneous across disciplinary, institutional, and experiential dimensions. Subsequent research can adopt longitudinal tracking designs to capture temporal dynamics in adoption behavior and incorporate new variables, such as algorithm transparency, susceptibility to hallucination, and ethical concerns, to extend the theoretical scope and explanatory rigor of BIs. Moreover, cross-group and cross-platform comparative studies can be conducted to more comprehensively reveal the dynamic laws of technology adoption, thereby strengthening the empirical foundation for sustainable, pedagogically coherent integration of GAI in higher education.

## 7 Conclusion

This study draws on a rigorously screened sample of 334 university students and employs a validated questionnaire, combined with SEM, to test the proposed theoretical framework. All seven research hypotheses received robust empirical support. Results indicate that PEU exerts significant positive effects on PU, ST, and UT; likewise, PU significantly predicts both ST and UT. ST, in turn, strongly enhances UT, which emerges as the most potent direct predictor of BI to use GAI platforms. ST and UT operate as sequential mediators—forming a theoretically grounded chain mediation pathway: “PEU → PU → ST → UT → BI.” This study extends the TAM’s applicability to GAI adoption in higher education contexts and offers actionable, evidence-based insights for refining platform design and integrating pedagogy.

## Data Availability

The raw data supporting the conclusions of this article will be made available by the authors, without undue reservation.
